# Case 5/2016 - A 56-Year-old Man Hospitalized for Unstable Angina, who Presented Recurrence of Precordial Pain and Cardiac Arrest with Pulseless Electrical Activity

**DOI:** 10.5935/abc.20160175

**Published:** 2016-11

**Authors:** Desiderio Favarato, Paulo Sampaio Gutierrez

**Affiliations:** Instituto do Coração (InCor) HC-FMUSP, São Paulo, SP - Brazil

**Keywords:** Chest Pain, Angina, Unstable, Myocardial Infarction, Heart Arrest, Pulse Wave Analysis

A 56-year-old man was admitted for a diagnostic evaluation of recent onset of chest pain.

Three days before admission, he had woken up in the middle of the night with a tight precordial pain of strong intensity, lasting for 20 minutes, radiating to the upper left limb and accompanied by dyspnea, which led him to seek medical attention. The myocardial injury markers were not increased, and the ECG was not considered suggestive of acute myocardial ischemia. After receiving a prescription of atenolol and aspirin, the patient was instructed to seek the Cardiology Department for outpatient care. In the days following the initial clinical presentation, he had two new episodes with lower intensity and sought medical assistance at this Hospital.

He also complained of dyspnea on exertion, which progressed over many years and had not intensified in the period. He was a smoker of 40 cigarettes per day and had known hypertension, which was controlled without medication.

On physical examination (on March 25, 2009), the patient was in good general condition, ruddy, hydrated, eupneic, with a regular heart rate of 80 bpm, blood pressure of 132 x 78 mmHg and normal pulmonary and heart auscultation. There were no changes upon examination of the abdomen. Peripheral pulses were normal, and there was no edema or signs of deep vein thrombosis.

The ECG at rest (on March 25, 2009) showed a sinus rhythm, a heart rate of 82 bpm, a PR interval of 160 ms, QRS duration of 110 ms, QT of 360 ms, left atrial enlargement, an ST segment flat in leads II, III and aVF and also elevated from V_1_ to V_3_ (increased convexity in V_1_ and increased concavity in V_2_ and V_3_), and positive and symmetrical T waves in leads V_5_ and V_6_ (Figure 1).

**Figure 1:**
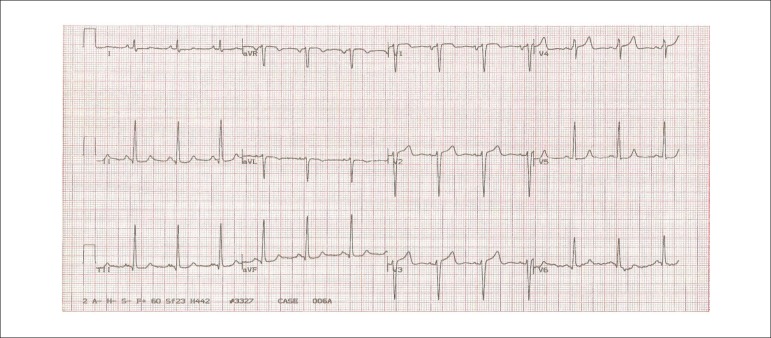
ECG (on March 25, 2009): sinus rhythm, left atrial enlargement, intraventricular conduction disturbance of the stimulus (dQRS = 110 ms), left ventricular enlargement, ventricular repolarization abnormalities (ST segment with a slight elevation from V_1_ to V_3_ and flat in the inferior leads), and symmetrical T waves in V_5_ and V_6_.

A stress test performed on the same day revealed during rest a heart rate of 83 bpm and blood pressure of 140 x 90 mmHg. The maximum heart rate achieved with 3 minutes of exercise was 127 bpm and the blood pressure measured at that moment was 140 x 90 mmHg. The test was interrupted due to physical fatigue, and at 4 minutes and 21 seconds, his heart rate returned to 87 bpm and blood pressure to 140 x 80 mmHg.

The ECG at peak exercise revealed a horizontal segment depression of 2 mm in V_5_ and of 1 mm in V_6_ (Figure 2), and the ECG during recovery showed an increase in the segment depression, which became descendant in the same leads and in the II, III and aVF leads (Figure 3).

**Figure 2:**
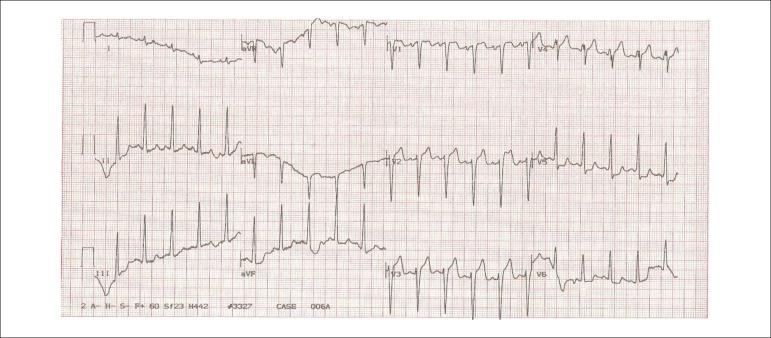
ECG (peak of exercise): horizontal segment depression of 2 mm in V_5_ and 1 mm in V_6_. Compatible with an ischemic response.

**Figure 3:**
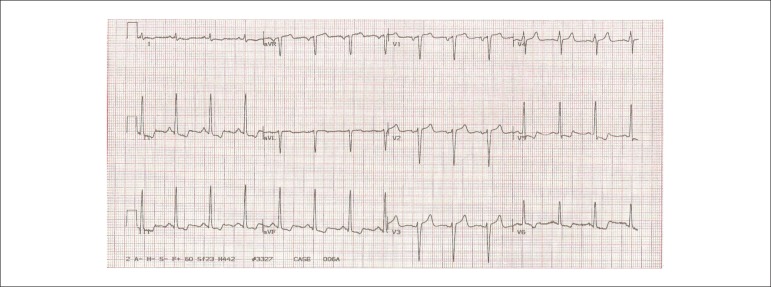
ECG (recovery): increase in segment depression in V_5_ and V_6_ and descending ST in the inferior leads. Compatible with extensive myocardial ischemia.

The test was considered positive for myocardial ischemia, and the patient was admitted for coronary cineangiography.

The laboratory tests (on March 25, 2009) revealed a hemoglobin of 15 g/dL, hematocrit 44%, leukocytes 8,000/mm³ (with normal differential count), platelets 142,000/mm³, glucose 109 mg/dL, urea 25 mg/dL, creatinine 0.72 mg/dL, total cholesterol 184 mg/dL, HDL-cholesterol 57 mg/dL, LDL-cholesterol 116 mg/dL, triglycerides 54 mg/dL, sodium 136 mEq/L, potassium 4.1 mEq/L, thrombin time (INR) 1.2, activated partial thromboplastin time (rel) 1.08, CK-MB 2.12 ng/mL, and troponin < 0.2 ng/mL.

The patient received a daily prescription of aspirin 200 mg, propranolol 120 mg, captopril 37.5 mg, simvastatin 40 mg, and enoxaparin 60 mg.

Coronary cineangiography (on March 26, 2009) revealed a left main coronary artery free of obstructions, anterior interventricular branch with an 80% obstructive lesion in the middle third, first diagonal with a 90% lesion in the ostium, and second diagonal with a 70% lesion in the ostium. There were multiple lesions in the circumflex branch, the largest with 90% in the middle third, the first and second left marginal branches with a 90% lesion, the third left marginal with a 60% lesion, and two posterior ventricular branches of the circumflex with irregularities. The right coronary artery was occluded and received grade 3 collaterals from the left coronary artery branches. The left ventricle was dilated, with accentuated diffuse hypokinesia and an apical dyskinetic area, with a filling failure suggestive of an apical thrombus.

Coronary artery bypass graft surgery was indicated.

About 6 hours after the coronary cineangiography, the patient developed dyspnea with bronchospasm and fenoterol 2 mg and ipratropium bromide 0.5 mg were administered by inhalation and hydrocortisone 100 mg intravenously. Propranolol and captopril were suspended.

Plasma level of CK-MB was 2.91 ng/mL.

A few hours later (at 1:45 a.m. on March 27, 2009), the patient presented cardiac arrest in asystole, for which external cardiac massage was started, three doses of 1 mg epinephrine and three doses of 0.5 mg atropine were administered, and endotracheal intubation was performed. The patient progressed with pulseless electrical activity, followed by ventricular fibrillation reverted with a 360-J shock. ECG tracing in a single lead revealed an extreme ST segment elevation (greater than 10 mm) (Figure 4) and, soon after, pulseless electrical activity with extreme bradycardia (25 bpm), irreversible asystole and death (at 2:30 a.m. on March 27, 2009).

**Figure 4:**
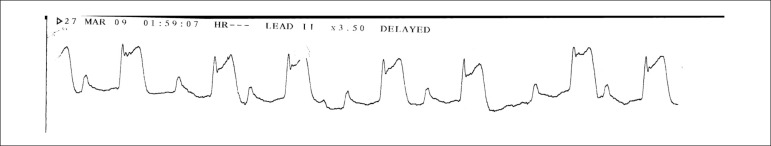
ECG (monitor strip, lead II): presence of a large ST segment elevation with a positive T wave. Suggestive of acute myocardial infarction.

## Clinical aspects

This is a 56-year-old man with a recent onset of angina and three-vessel lesions on coronary cineangiography, who presented with dyspnea and, a few hours later, cardiorespiratory arrest with intense ST segment elevation.

Angina is the initial manifestation in half of the patients with coronary artery disease. Even when stable, *i.e.*, without changes in the triggering events or intensity, a duration of the episodes longer than 3 months doubles the risk of cardiovascular events.^1,2^

Unstable angina is defined by the presence of at least one of the following findings: occurring at rest or upon exertion, lasting more than 20 minutes, or having a progressive pattern (more intense, prolonged, or frequent).^3^

Thus, the anginal pattern presented by the patient was that of unstable angina, which implies a 6% risk of death and of requirement of revascularization in the first year.^4^

The occurrence of unstable angina, such as that of the patient, is greater in men than in women and increases with age, reaching its peak frequency at the age of 70 years and decreasing in the following decades.^5^

According to unstable angina risk classifications, the patient would be classified with TIMI 2 and GRACE 86 scores, which predict risks of death at 30 days of 5.4% and 4%, respectively.

The risk of the patient was been stratified with the stress test, which proved positive with a frequency of 156 bpm but showed an increased segment depression during recovery, which increased his odds of being a multivessel patient or carrier of a left main coronary artery lesion. This was confirmed by coronary cineangiography, which revealed critical lesions in the anterior, diagonal, circumflex, and marginal interventricular arteries, as well as occlusion of the right coronary artery.

Autopsy studies show that 75% of the fatal infarctions have plaque rupture, while the remaining 25% have endothelial erosion.^6^

*In vivo* studies using optical coherence tomography have shown a similar or slightly increased number attributed to erosion in patients with unstable angina (from 22% to 31%).^7,8^

More recently, the erosion of the atherosclerotic plaque has been more associated with thrombosis in infarctions either with or without segment elevation, which led to the questioning of the vulnerable plaque concept (plaques with a thin fibrous cap with less than 65 micra, and rich lipid core.)^9^

As for the final event, cardiorespiratory arrest with ECG during recovery showing QRS with monophasic R wave, large segment elevation with J point elevation, and positive T wave (the so-called complex M in acute myocardial infarction) has been associated with the presence of left ventricular free wall rupture, but without much specificity.^10^

It is known that the occurrence of hypotension and bradycardia followed by pulseless electrical activity are typical signs of bleeding to the pericardial sac; additionally, pulseless electrical activity in the absence of previous heart failure in a patient in a first heart attack has a predictive accuracy of 97.6% for the diagnosis of left ventricular free wall rupture.^11^ (**Dr. Desiderio Favarato**)

**Diagnostic hypothesis:** ischemic heart disease unstable angina followed by acute myocardial infarction with likely left ventricular free wall rupture. (**Dr. Desiderio Favarato**)

## Necropsy

**Figure 5:**
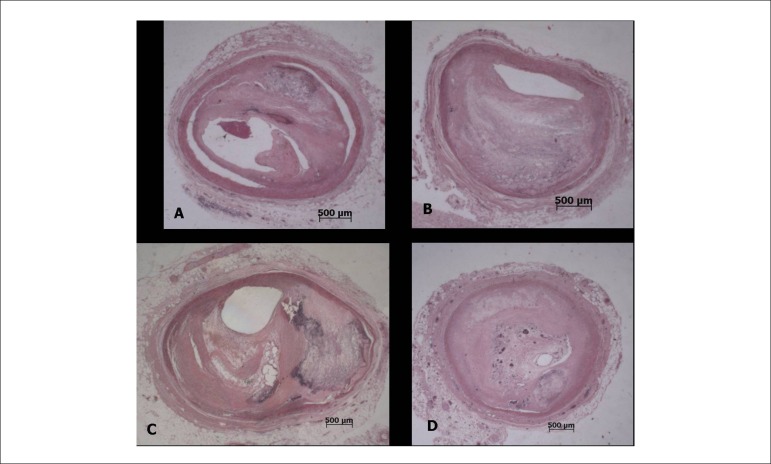
Panel showing histological sections of the coronary arteries with the highest degree of obstruction. DA4 - 4^th^ centimeter of the anterior interventricular branch; Cx4 - 4^th^ centimeter of the circumflex branch: MEII1 - initial segment of the second left marginal branch; CD10 - 10^th^ centimeter of the right coronary artery, with a thrombus undergoing organization. Stained with hematoxylin and eosin, objective magnification=1X.

**Figure 6:**
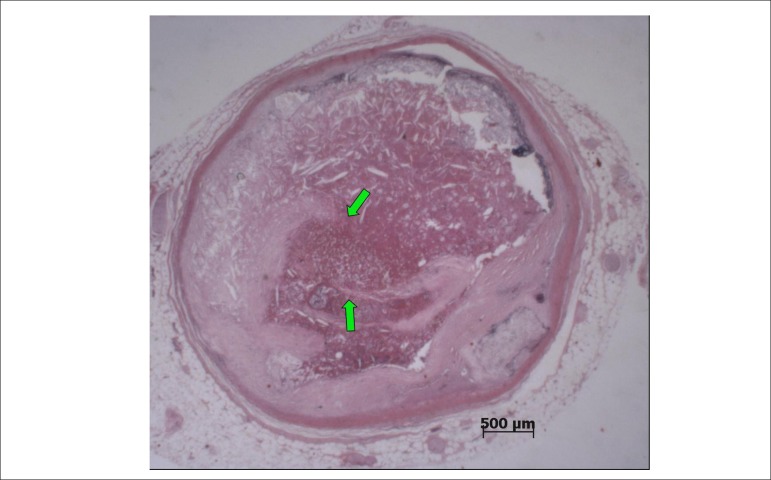
Histological section of the 6^th^ centimeter of the right coronary artery, with atherosclerotic plaque rupture (arrows) and a recent occlusive thrombosis. Stained with hematoxylin and eosin, objective magnification=1X.

**Figure 7:**
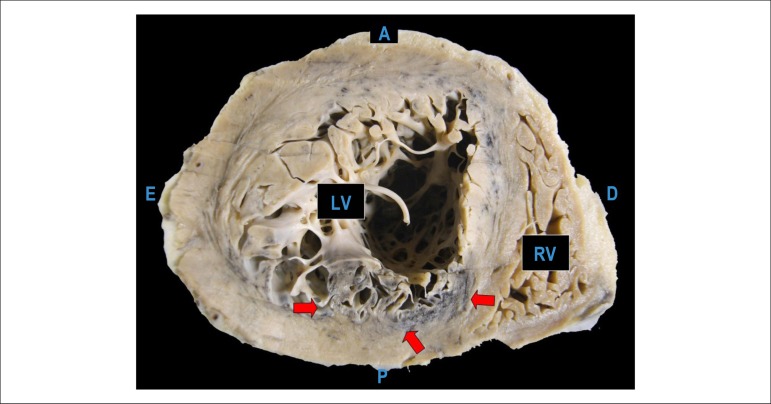
Cross section of the heart showing the infarcted myocardial area undergoing organization, with grayish-white color, in the rear wall (inferior, diaphragmatic) of the left ventricle (arrows). A - anterior surface of the heart; D - right side of the heart; E - left side of the heart; P - rear surface (inferior, diaphragmatic) of the heart.

**Figure 8:**
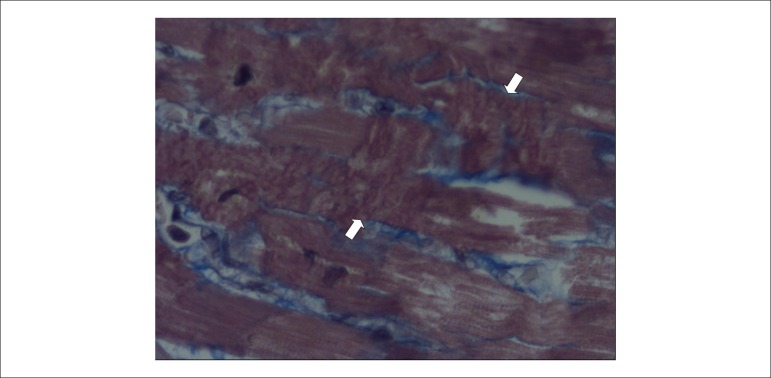
Histological section of the posterior (inferior, diaphragmatic) wall of the left ventricle showing areas of recent necrosis (contraction bands, between arrows). Masson's trichrome stain, objective magnification=40X.

The necropsy evidenced systemic atherosclerosis as the main disease, with severe involvement of the aorta and coronary arteries, with an obstruction greater than 80% in the three main branches (Figure 5). In the right coronary artery, there was a thrombus undergoing organization in the distal bed, and a recent, occlusive thrombosis with plaque rupture at the 2nd, 3rd, and 6th centimeters (Figure 6). Due to these thromboses, in the posterior wall (inferior, diaphragmatic) of the left ventricle, there were a small infarction in the final stage of organization (Figure 7) and morphologically dubious areas of myocardial infarction with a few hours of onset (Figure 8); the latter change was also present in the lateral wall of the left ventricle, in the subendocardial region of the remaining walls of this chamber, and in the right ventricle. As a possible consequence of a recent infarction, there was acute pulmonary edema.

Other necropsy findings corresponded to conditions associated with atherosclerosis - systemic arterial hypertension (benign nephrosclerosis and concentric left ventricular hypertrophy) and smoking (chronic obstructive pulmonary disease). (**Dr. Paulo Sampaio Gutierrez**)

**Anatomopathological diagnosis:** coronary atherosclerosis.

***Cause of death***: acute pulmonary edema. (**Dr. Paulo Sampaio Gutierrez**)

## Comments

This patient had severe triple-vessel coronary atherosclerotic obstructions. In the past, he had thrombosis of the right distal coronary artery bed, with infarction in its territory of irrigation. Subsequently, he had new thrombosis in this artery, at a more proximal level, which possibly determined the myocardial infarction, thus causing the patient's death. It should be emphasized, however, that the morphological aspect was highly suggestive (but not indisputable) of a recent infarction. For this reason, the precise extent of the ischemic injury could not be determined. The presence of acute pulmonary edema strengthens the possibility of the occurrence of the recent infarction. (**Dr. Paulo Sampaio Gutierrez**)

**Section Editor:** Alfredo José Mansur (ajmansur@incor.usp.br)

**Associated Editors:** Desidério Favarato (dclfavarato@incor.usp.br), Vera demarchi Aiello (anpvera@incor.usp.br)
